# Effect of simvastatin on castration-resistant prostate cancer cells

**DOI:** 10.1186/1476-511X-13-56

**Published:** 2014-03-26

**Authors:** Jenny Hanbi Kim, Michael E Cox, Kishor M Wasan

**Affiliations:** 1 Faculty of Pharmaceutical Sciences, University of British Columbia, 2405 Wesbrook Mall, Vancouver, British Columbia, Canada; 2 Department of Urologic Sciences, The Prostate Centre at Vancouver General Hospital, 2660 Oak Street, Vancouver, British Columbia, Canada

**Keywords:** Simvastatin, Castration-resistant prostate cancer, HMGCR, Cholesterol synthesis

## Abstract

**Background:**

In castration-resistant prostate cancer (CRPC), recent evidence has demonstrated the persistence of the intratumoral androgens. The multi-step androgen synthesis pathway originates from cholesterol, which can be obtained by cells from several major sources including intracellular synthesis through an enzyme 3-hydroxy-3-methylglutaryl-coenzyme A reductase (HMGCR). The inhibition of this enzyme by the use of statins has been investigated in prostate cancer as a possible therapeutic target for blocking the de novo androgen synthesis resulting in decreased tumor growth. However, the effectiveness of statins in CRPC has not been investigated.

**Methods:**

Castration-resistant C4-2 and androgen-sensitive LNCaP cells were treated with Simvastatin for 48 hours. Dose-dependent responses to Simvastatin were analyzed using cell proliferation and cytotoxicity assays. Cellular growth curve was generated using haemocytometer. HMGCR activity was assessed using ^14^C-acetic acid detected by thin layer chromatography, and the protein expression was quantified using western blot analysis. Intracellular cholesterol and prostate specific antigen (PSA) levels were quantified using enzyme-linked immunosorbent assays (ELISA).

**Results:**

Significant decrease in cell viability and growth curve observed at 75 μM of Simvastatin compared to no treatment group in the castration-resistant C4-2 cells. HMGCR activity was significantly decreased up to 50% and 70% at 50 μM and 75 μM of Simvastatin respectively compared to the vehicle control in C4-2 cells. Simvastatin did not affect the protein expression. 80% decrease in the amount of total intracellular cholesterol levels was observed in 75 μM Simvastatin treatment group compared to vehicle control. PSA secretion levels were significantly reduced in the C4-2 cell line at 50 μM and 75 μM of Simvastatin compared to vehicle control.

**Conclusion:**

The inhibition of HMGCR via Simvastatin lowered the viability of castration-resistant C4-2 cells. Simvastatin’s ability to limit the endogenous supply of cholesterol contributes to the effects seen in cell viability.

## Background

Prostate cancer (PCa) occurs in the form of an unregulated cellular growth in the prostate, a gland in the male reproductive system, leading to symptoms such as erectile dysfunction, hematuria, and pain. PCa is commonly diagnosed in men over the age of 50, after which incidence rates increase with age; it is the most common cancer diagnosed among North American men, accounting for 28% of all cancers diagnosed, and is the second most common cause of cancer death among the same cohort (10%) in 2012 [1,2]. Upon diagnosis, local PCa is treated with surgical removal of the tumor, radiation therapy, and/or androgen deprivation therapy (ADT). Within the prostate gland, androgens such as testosterone and dihydrotestosterone (DHT) promote cell growth and proliferation via androgen receptor (AR), a ligand-responsive transcription factor. Upon ADT, 99% of prostate cancer patients reach 5-year survival due to apoptotic regression of tumor from the lack of androgen growth stimuli [3,4]. However, patients with metastatic prostate cancer are subject to temporary remission. Eventually the tumor regrows despite the depleted level of circulating androgens that is insufficient to support the prostate tumor growth; this is referred to as castration-resistant prostate cancer (CRPC) [3,4].

Previously CRPC has been known as an ‘androgen independent’ cancer due to its ability to grow and metastasize despite the low androgen environment. Recent findings by multiple groups however have shown that CRPC cells have the ability to produce their own intracellular androgens that promote growth via AR activation without having to rely on exogenous androgen supply – known as intratumoral *de novo* steroidogenesis [5,6]. Androgen levels within metastastic tumors of castrated men are found to be higher than the levels within the primary prostate cancer tumors in untreated men [7]. In addition, CRPC tumors are shown to continuously express the necessary enzymes to create androgens intracellularly [8,9]. While the steps towards androgen production consist of multiple pathways, all the steps originate from a common upstream precursor molecule, cholesterol [10].

Cellular cholesterol homeostasis is comprised of complex and multiple regulatory pathways as cholesterol has important functions in humans including regulating membrane fluidity, influencing cellular signaling, and being a precursor for bile and androgens [11]. Cells obtain cholesterol from two major sources: exogenous and endogenous supplies. Exogenous cholesterol supply involves uptake of cholesterol from circulating lipoproteins via membrane transporters such as Scavenger Receptor Class B Type I (SR-BI) and low density lipoprotein receptor (LDLr). Once in the cell, cholesterol is stored as cholesteryl esters in lipid droplets and metabolized accordingly to cell’s demand for cholesterol via acetyl-CoA acyltransferase (ACAT) and hormone sensitive lipase (HSL). Endogenously, cholesterol is synthesized from acetyl-CoA in the endoplasmic reticulum through is the mevalonate pathway, in which the rate-limiting is step 3-hydroxy-3-methylglutaryl-coenzyme A reductase (HMGCR) that is responsible for converting 3-hydroxy-3-methylglutaryl-coenzyme A (HMG-CoA) into mevalonate [11,12]. Mevalonate molecule further undergoes multiple reactions to be converted into cholesterol downstream.

While normal physiological cholesterol homeostasis is tightly regulated, it has been shown that this process is dysregulated in CRPC, suggesting a constant, unregulated supply of cholesterol to meet cellular requirements including supply for *de novo* steroidogenesis [6,13,14]. A potential site of dysregulation has shown to be at site of cholesterol uptake via SR-BI; our group has shown that SR-BI protein expression was significantly increased upon progression to castration-resistance in the LNCaP xenograft model [8]. Also, upon inhibition of cholesterol uptake via SR-BI silencing *in vitro*, a compensatory cholesterol synthesis via increased HMGCR activity was identified and significant decreases in prostate specific antigen (PSA) and cell viability of CaP cells were observed [15]. However, relevant changes in total cholesterol concentration and androgen levels were not seen, most likely due to the activation of compensatory cholesterol synthesis via HMGCR [15]. Recently, our group has demonstrated that HMGCR expression and activity, and thereby cholesterol synthesis, was increased during CRPC progression in LNCaP xenografts [6,16]. Statins, inhibitors of HMGCR, have been the subject of many PCa studies with mixed results [17-19]. Recently, an association between statin users and a reduction in the onset of the aggressive, late-stage disease state was reported and has been paired with decreased serum androgens [20]. However to date, only a handful of research has looked directly at the effect of statin as a treatment option for CRPC; it has been shown that inhibition of cholesterol synthesis via statin prevents cell proliferation by inducing apoptosis through reduction in nuclear factor-κB activity [21]. However there are limitations to the studies with regards to cell line specificity, and the lack of cholesterol metabolism data.

The present study sought to determine the role of HMGCR in castration-resistant prostate cancer cells in terms of cell viability, cholesterol synthesis, and PSA production when the cholesterol synthesis via HMGCR is blocked with Simvastatin. LNCaP and C4-2 cells share similar features by expressing AR and producing PSA; C4-2 cells are lineage-derived second generation subline of LNCaP cells that were derived by subcutaneous co-inoculation of LNCaP and osteosarcoma cells in castrated mice [22]. Therefore, LNCaP cells were selected as the appropriate control cell line.

## Results

### C4-2: castration-resistant prostate cancer cell line

#### *Cytotoxicity of simvastatin treated C4-2 cells*

**Figure 1:**
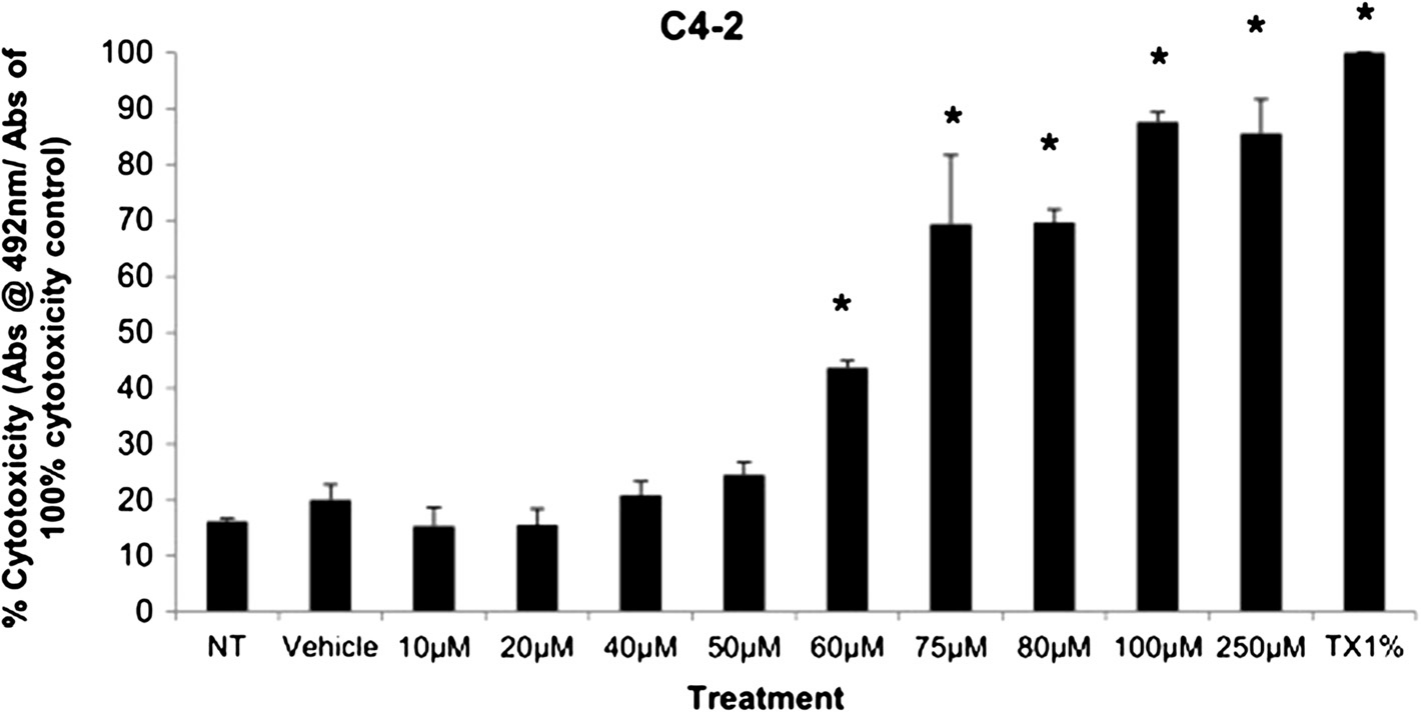
**Cell cytotoxicity is demonstrated as % cytotoxicity based on a 100% cytotoxic control, representing the amount of LDH in the media of C4-2 cells treated with increasing dose of Simvastatin.** Columns, mean (n = 5); bars, ±SEM. *P < 0.05, Vehicle control versus μM, 80 μM, 100 μM, 250 μM.

The cytotoxicity of cells was measured based on the presence of lactate dehydrogenase (LDH) in the media, which indicates poor cellular membrane integrity and cell lysis. LDH in media causes the conversion of a tetrazolium salt reagent into a red formazan product, which the absorbance can be measured at 492 nm. Results are expressed as values normalized to a 100% death control which represents untreated cells lysed with a 1% Triton X-100 solution. The means of the cytotoxicity measured at different Simvastatin doses were compared to the Vehicle Control, consisting of 0.5% DMSO. The means were found to be significantly different at 60 μM, 75 μM, 80 μM, 100 μM, and 250 μM in C4-2 cells (Figure 1).

#### *Cell viability and growth curve of simvastatin treated C4-2 cells*

**Figure 2:**
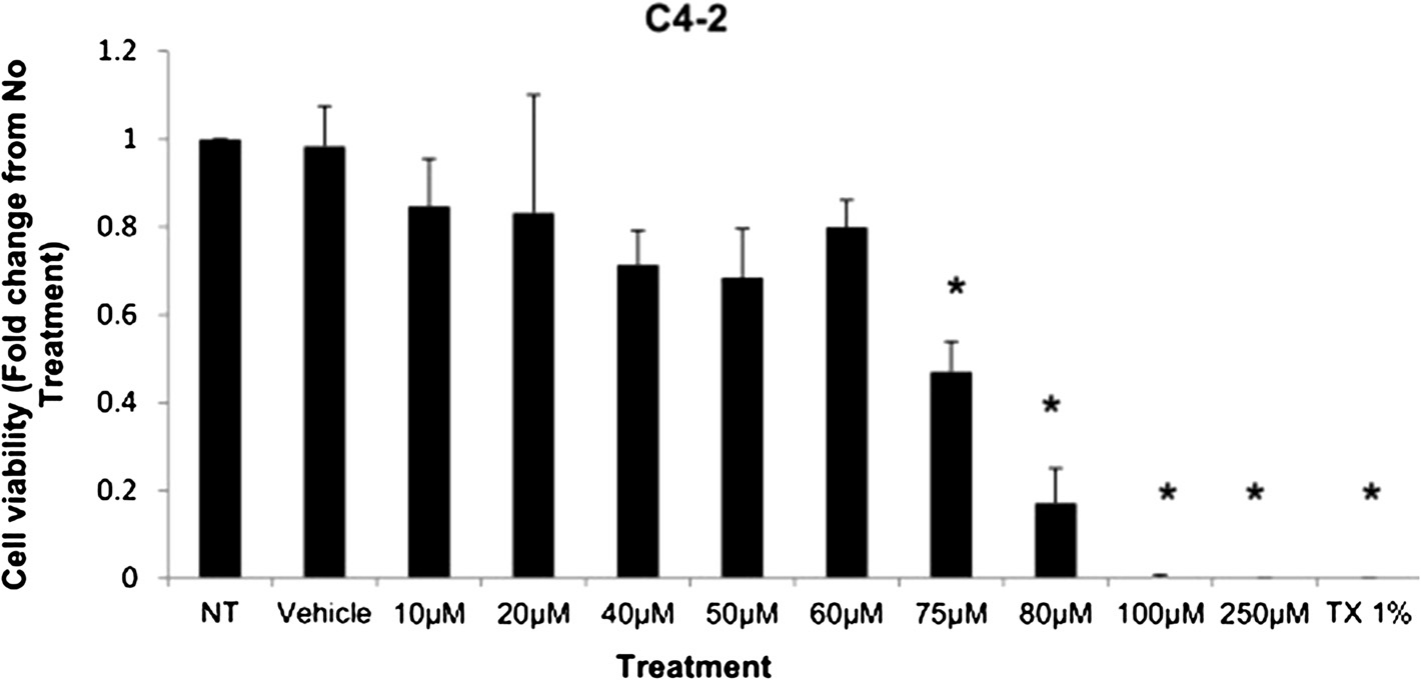
**Cell viability is demonstrated as fold change in viability from the negative control in C4-2 cells treated with increasing dose of Simvastatin.** Columns, mean (n = 5); bars, ±SEM. *P < 0.05, Vehicle control versus 60 μM, 75 μM, 80 μM, 100 μM, 250 μM and TX1%.

The cell viability was measured based on the presence is of dehydrogenase enzymes in metabolically active cells which are able to bioreduce the 3-(4,5-dimethylthiazol-2-yl)-5-(3-carboxymethoxyphenyl)-2-(4-sulfophenyl)-2H-tetrazolium (MTS) tetrazolium reagent into a colored formazan product that can be measured at 490 nm, producing signal measurements that are directly proportional to the number of living cells in culture. The viability of cells was normalized to protein levels (μg) in the respective cell samples. As shown in Figure 2, cellular viability, expressed as fold change from negative control, decreased significantly at 75 μM, 80 μM, 100 μM, 250 μM, and Triton-X 1% control for C4-2 cells in a dose-dependent manner.

**Figure 3:**
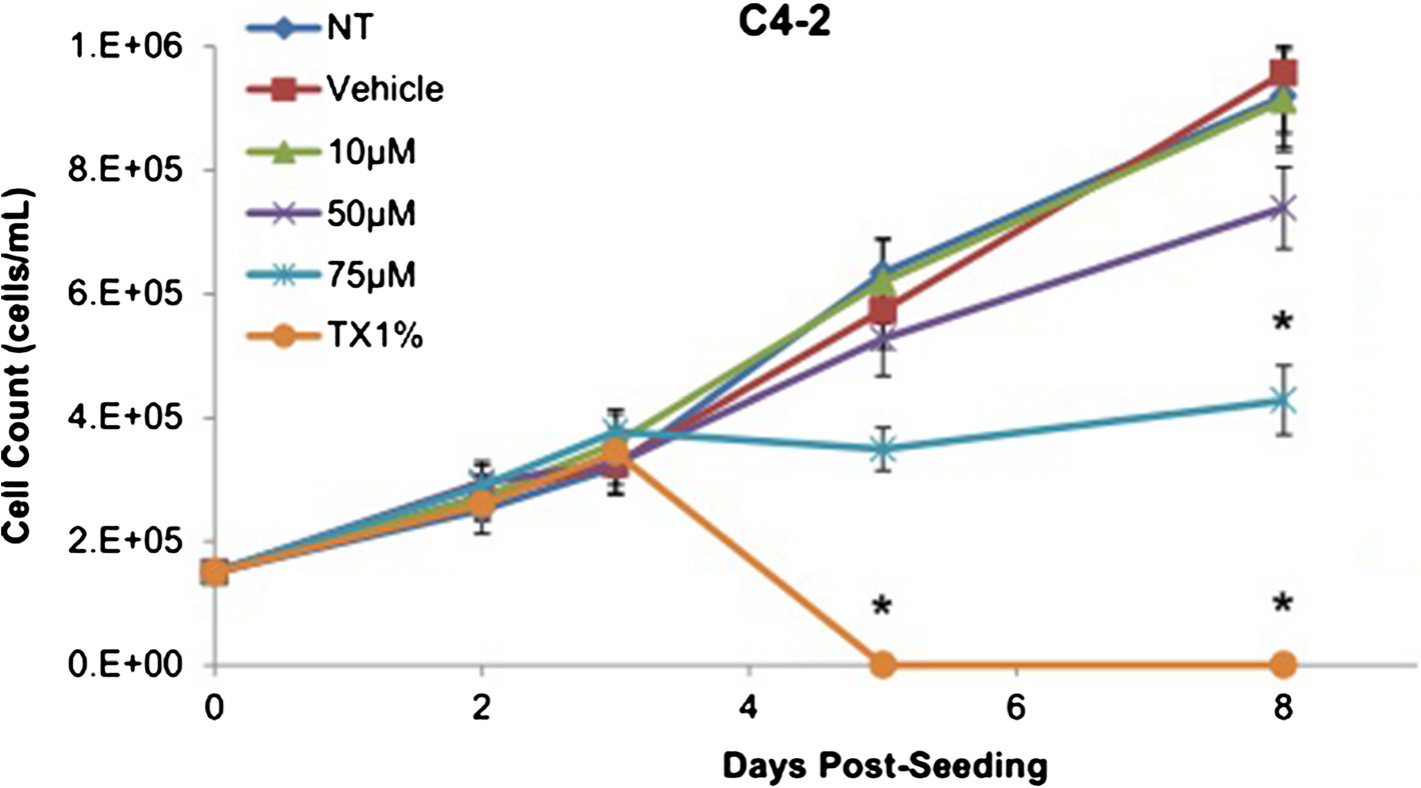
**Cumulative cell growth curve expressed as total cell count of C4-2 cells over 8 days.** Curve, mean (n = 5); error bars, ± SEM. *P < 0.05, Vehicle control versus TX1% in C4-2 at Day 5; Vehicle control versus 75 μM and TX1% at Day 8.

The cumulative growth curve was generated to observe the long-term cell growth patterns over 8 days post-seeding by measuring the total cell count using haemocytometer at 48 hours post-seeding (Day 2), before SV treatment on Day 3, 48 hours post-treatment (Day 5), and 72 hours post-Simvastatin removal (Day 8). As shown in Figure 3, the only significant difference in the growth pattern of C4-2 cells upon Simvastatin treatments compared to vehicle control was at 75 μM of Simvastatin at Day 8. Despite the difference in the growth rate of C4-2 cells at 75 μM compared to vehicle control at Day 5, the difference was not statistically significant.

#### *HMGCR activity and protein expression*

**Figure 4:**
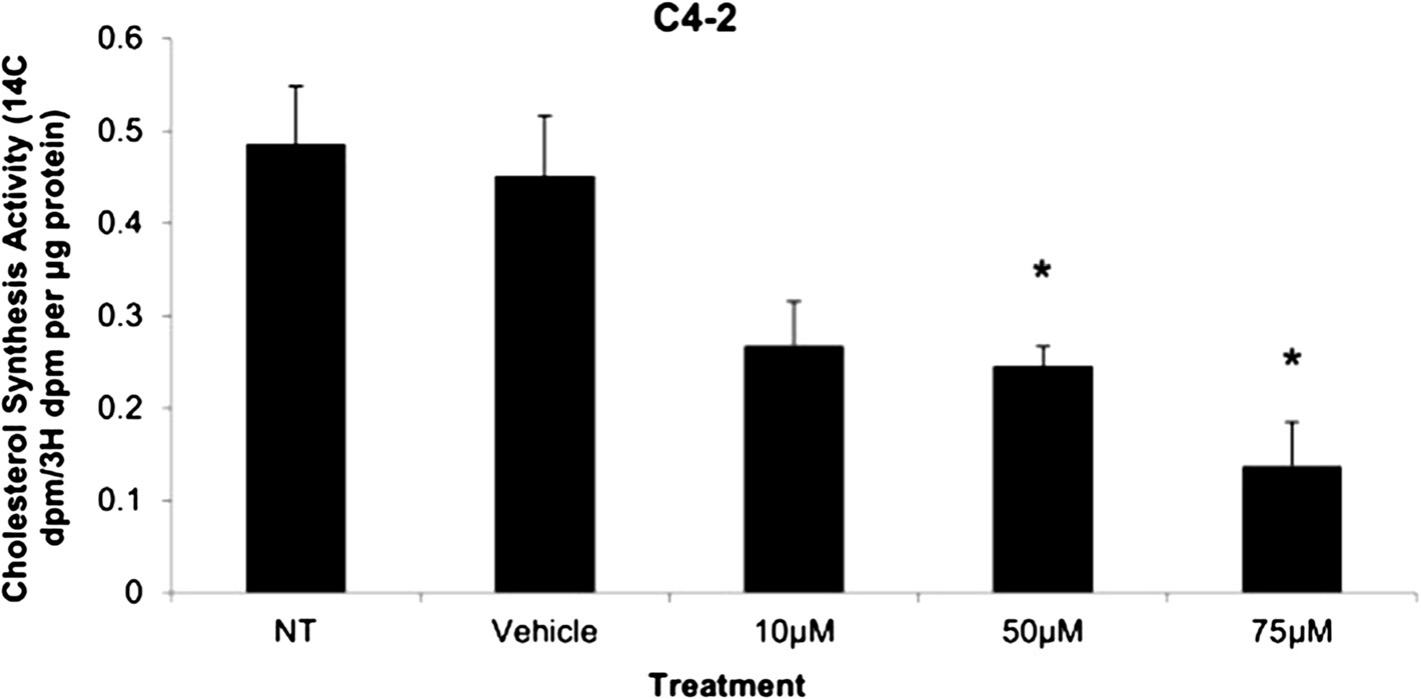
**Cholesterol synthesis measurement by incorporation of **^**14**^**C-acetic acid into cellular cholesterol as measured by β-scintillation counter in C4-2 after 48 hour Simvastatin treatment followed by 3 hour **^**14**^**C-acetic acid treatment and separation by thin layer chromatography.** Columns, mean (n = 5); bars, ±SEM. *P < 0.05, Vehicle Control versus 50 μM, and 75 μM.

β-scintillation readings of silica gel at a Rf value of approximately 0.25 indicated the location of cholesterol on the stationary phase as compared to positive cold cholesterol control sample. The measurements were used to assess the cholesterol synthesis activity via HMGCR enzyme by the incorporation of radiolabeled acetate precursor molecule into cholesterol synthesis pathway. The β-scintillation readings (^14^C DPM/^3^H DPM) were normalized to protein levels (μg) as measured by Lowry assay. The mean cholesterol synthesis activities in C4-2 cells significantly decreased up to 50% and 70% upon 50 μM and 75 μM of Simvastatin respectively compared to vehicle control group (Figure 4).

**Figure 5:**
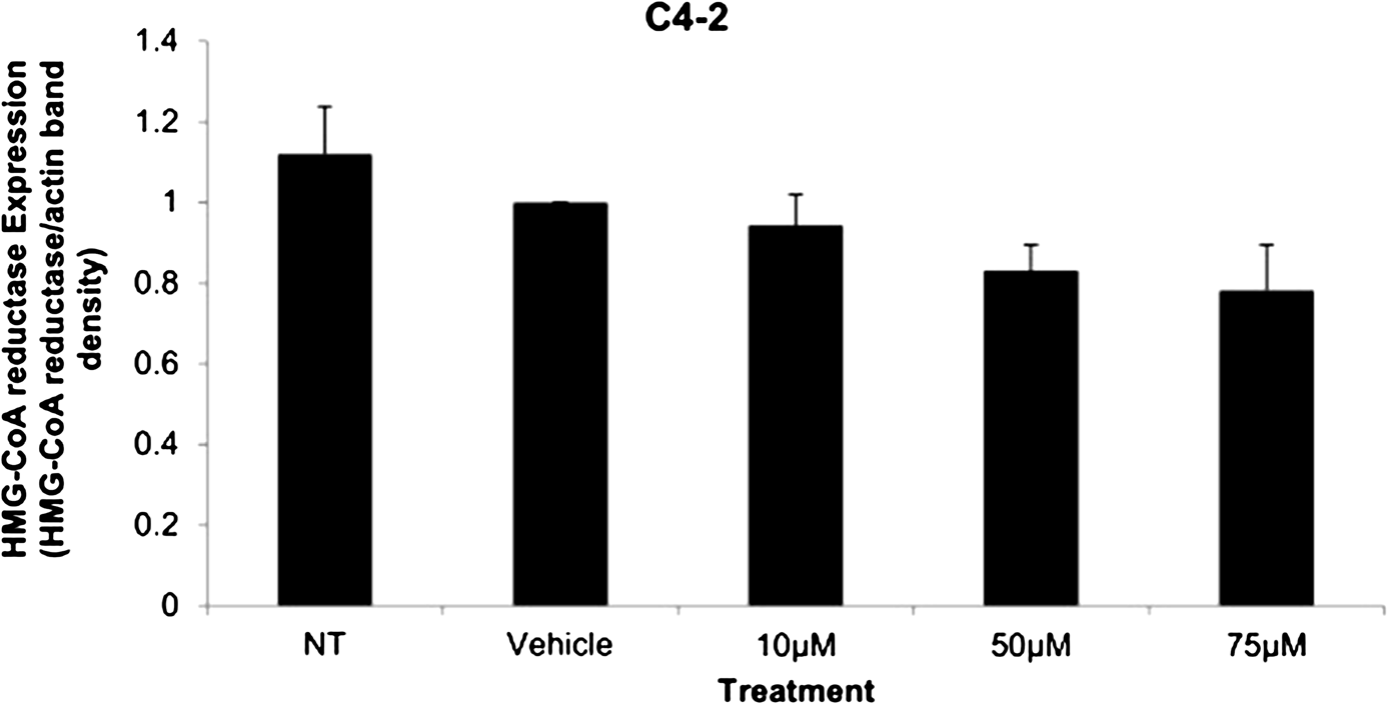
**Relative protein expression in C4-2 cells to vehicle control expression after 48 hour Simvastatin treatment, normalized to actin expression.** Columns, mean (n = 4); bars, ±SEM.

Western blot analysis of C4-2 cell lysates showed that there is no significant decrease in HMGCR expression upon Simvastatin treatments when compared to the vehicle control (Figure 5).

#### *Cellular cholesterol of simvastatin treated C4-2 cells*

**Figure 6:**
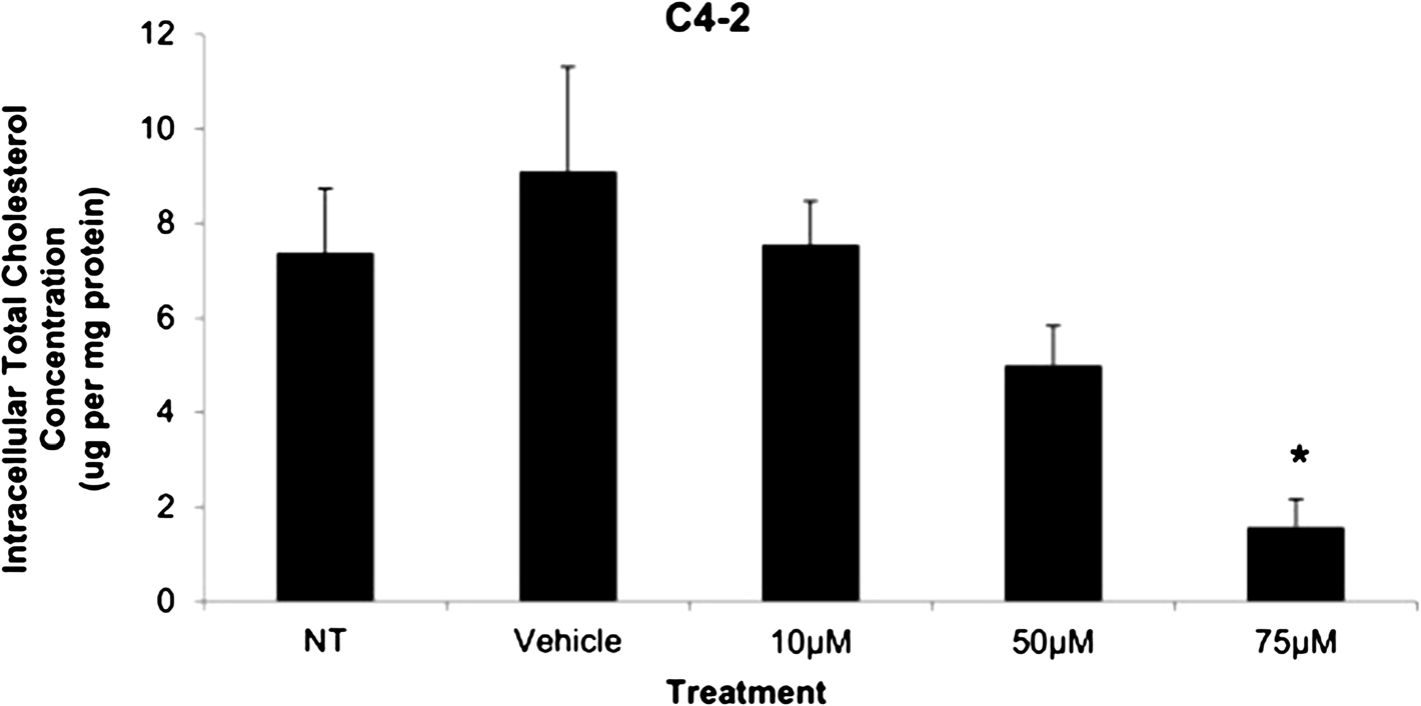
**Total cholesterol concentration in whole cell lysates of C4-2 cells after 48 hour Simvastatin treatment.** Columns, mean (n = 6); bars, ±SEM. *P < 0.05, Vehicle control versus 75 μM.

The total intracellular cholesterol concentrations were measured in C4-2 cells treated with Simvastatin for 48 hours, and the values were normalized to protein (mg) in the cell samples from the respective wells of whole cell lysates. Significant decrease in the amount of total cholesterol was observed at 75 μM of Simvastatin compared to vehicle control in C4-2 cells (Figure 6). The decrease in cholesterol levels corresponded to the decrease in HMGCR protein activity at 75 μM Simvastatin treatment results.

#### *PSA secretion is reduced in C4-2 cells treated with simvastatin*

**Figure 7:**
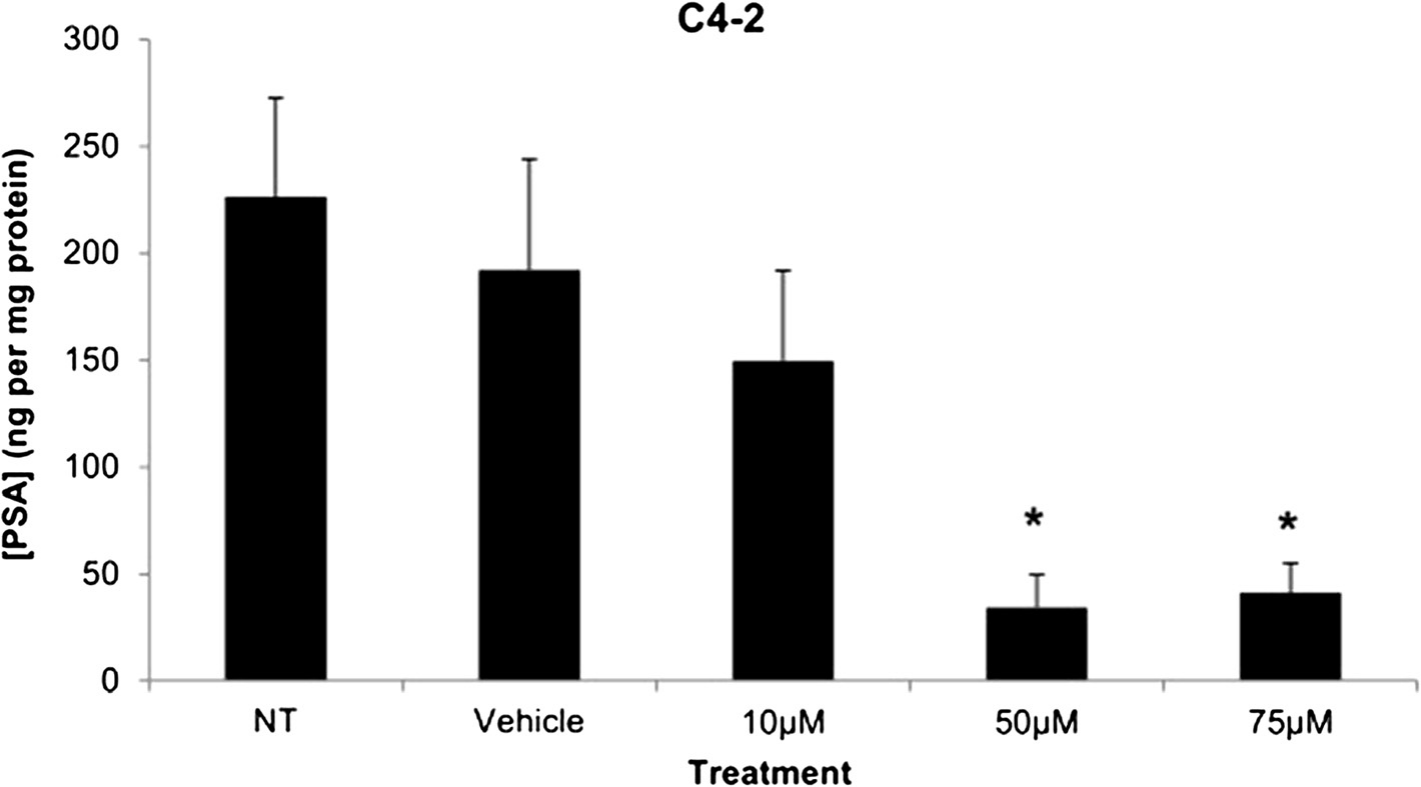
**PSA production demonstrated as secreted PSA levels in the media of C4-2 after 48 hour Simvastatin treatment.** Columns, mean (n = 6); bars, ±SEM. *P < 0.05, Vehicle control versus 50 μM and 75 μM.

The levels of PSA secretion of C4-2 cells were quantified in the supernatant or treated cells. The amount of PSA is expressed as concentration (ng/mL) and was then normalized to the amount of protein (mg) in the cell samples from the respective wells of the analyzed supernatant. The PSA secretion levels were significantly less at 50 μM and 75 μM of Simvastatin treatments compared to vehicle control in C4-2 cells; vehicle control had secreted 192.1 ± 52.0 ηg/mL per mg protein as compared to 34.4 ± 15.4 ng/mL per mg at 50 μM and 41.32 ± 14.0 ng/mL per mg at 75 μM of Simvastatin treatments (Figure 7).

#### *LNCaP: androgen-sensitive prostate cancer cell line*

**Figure 8:**
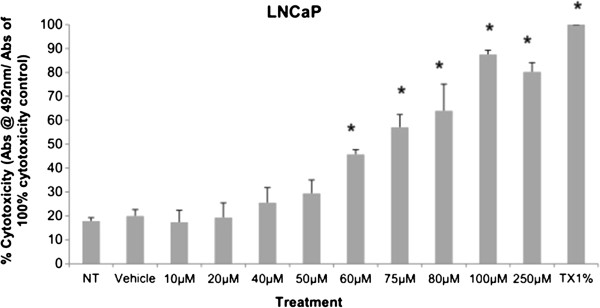
**Cell cytotoxicity is demonstrated as % cytotoxicity based on a100 % cytotoxic control, representing the amount of LDH in the media of LNCaP cells treated with increasing dose of Simvastatin.** Columns, mean (n = 5); bars, ±SEM. *P < 0.05, Vehicle control versus 75 μM, 80 μM, 100 μM, 250 μM and TX1%.

**Figure 9:**
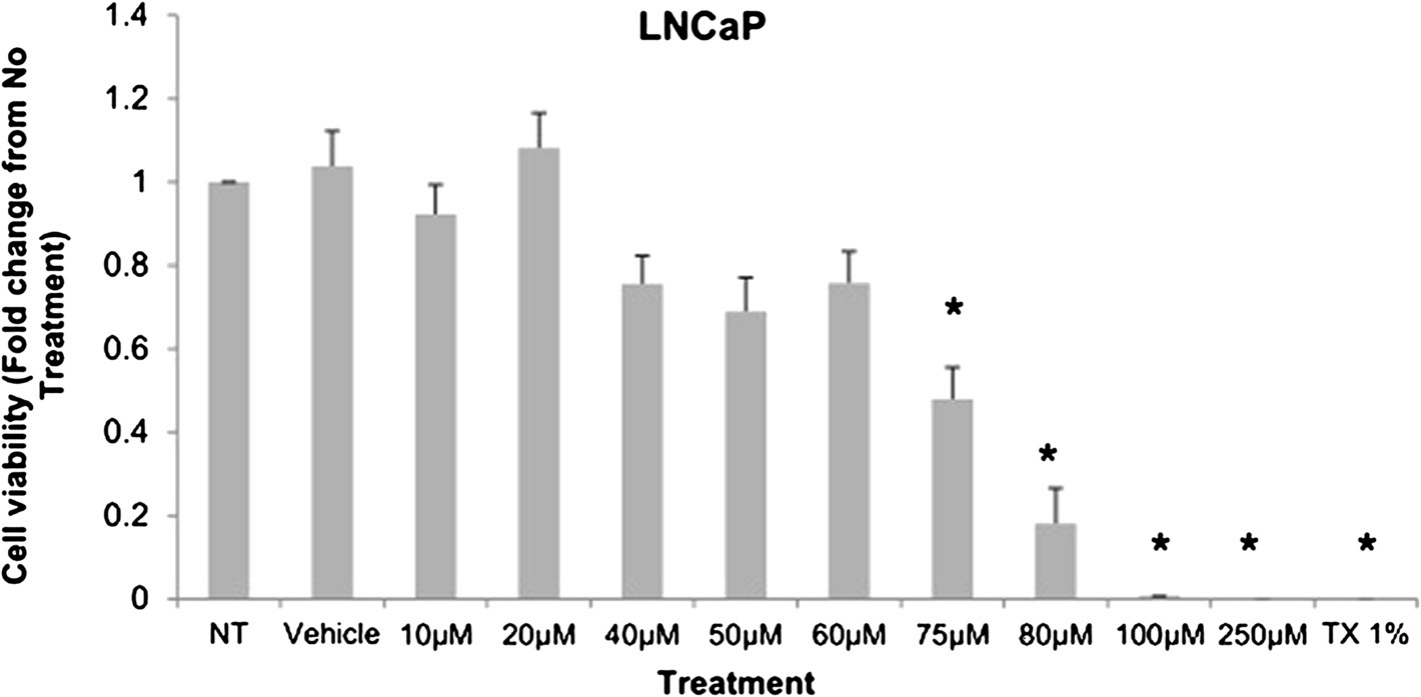
**Cell viability is demonstrated as fold change in viability from the negative control in LNCaP cells treated with increasing dose of Simvastatin.** Columns, mean (n = 5); bars, ±SEM. *P < 0.05, Vehicle control versus 60 μM, 75 μM, 80 μM, 100 μM, 250 μM and TX1%.

**Figure 10:**
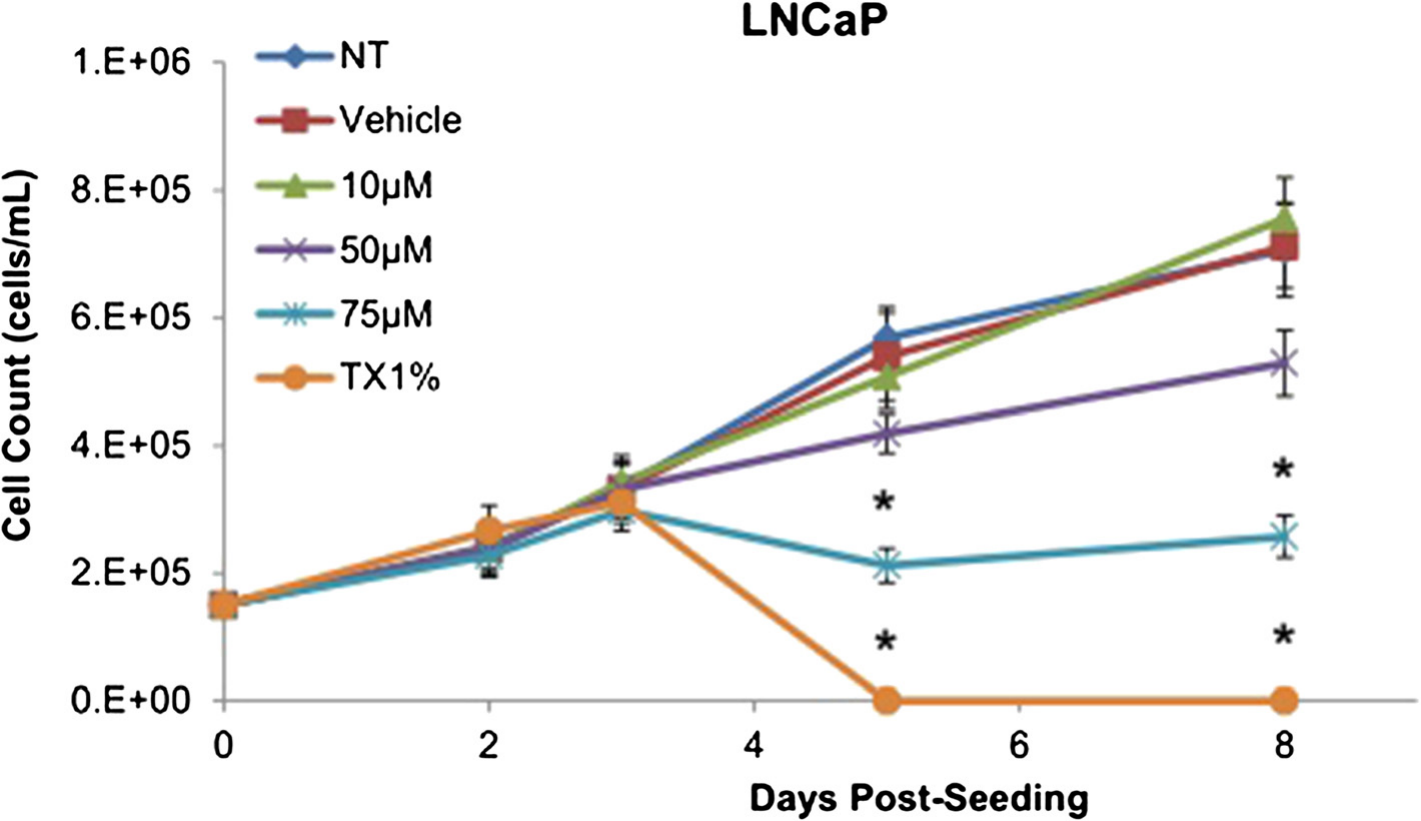
**Cumulative cell growth curve expressed as total cell count of LNCaP cells over 8 days.** Curve, mean (n = 5); error bars, ± SEM. *P < 0.05, Vehicle control versus 75 μM and TX1% in at Day 5 and 8.

**Figure 11:**
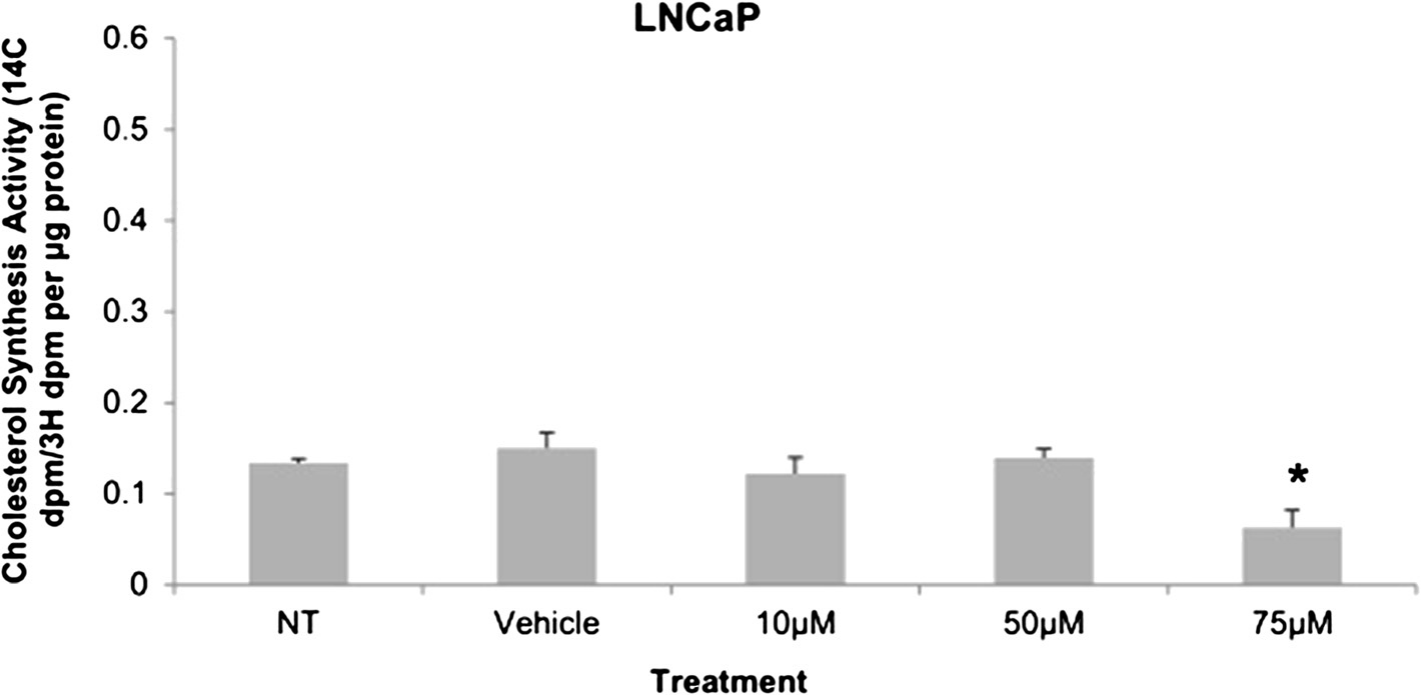
**Cholesterol synthesis measurement by incorporation of **^**14**^**C-acetic acid into cellular cholesterol as measured by β-scintillation counter in LNCaP after 48 hour Simvastatin treatment followed by 3 hour **^**14**^**C-acetic acid treatment and separation by thin layer chromatography.** Columns, mean (n = 5); bars, ±SEM. *P < 0.05, Vehicle control versus 75 μM.

**Figure 12:**
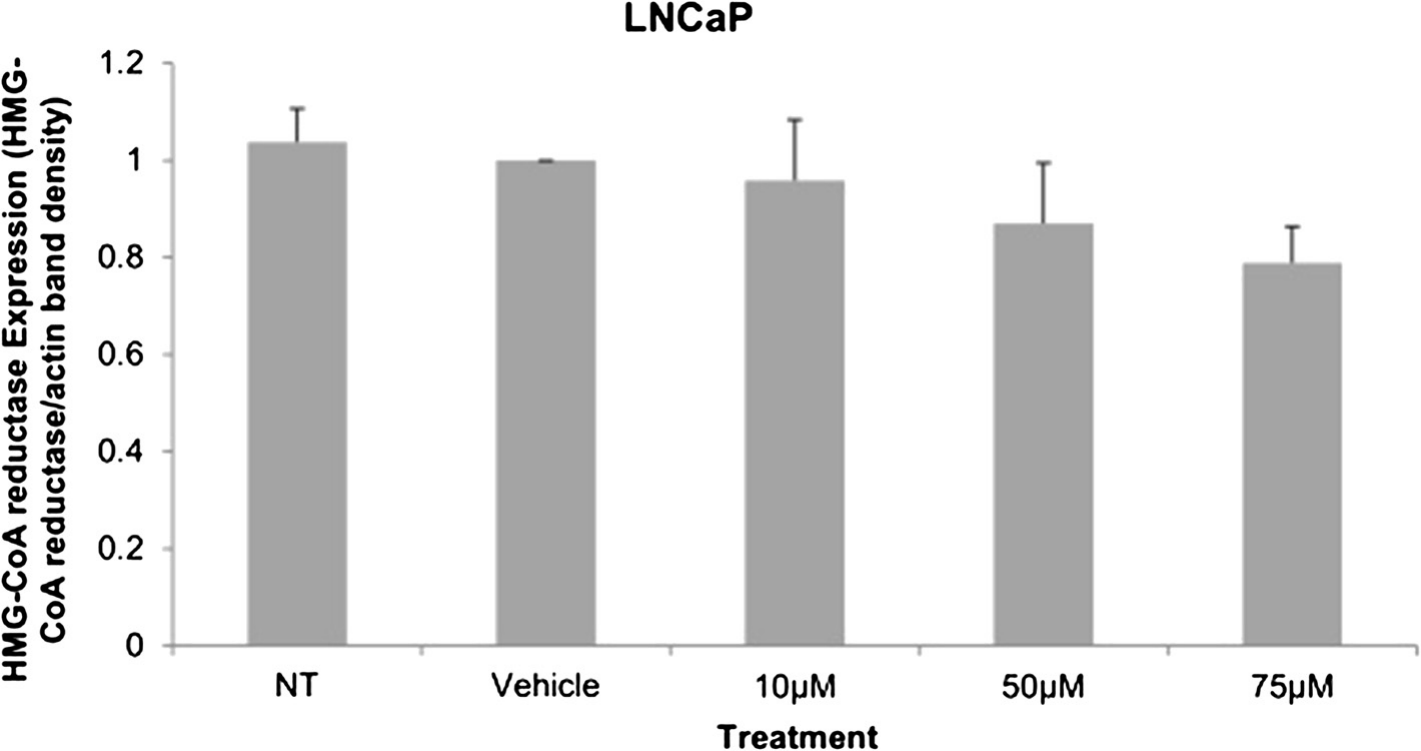
**Relative protein expression in LNCaP cells to vehicle control expression after 48 hour Simvastatin treatment, normalized to actin expression.** Columns, mean (n = 4); bars, ±SEM.

**Figure 13:**
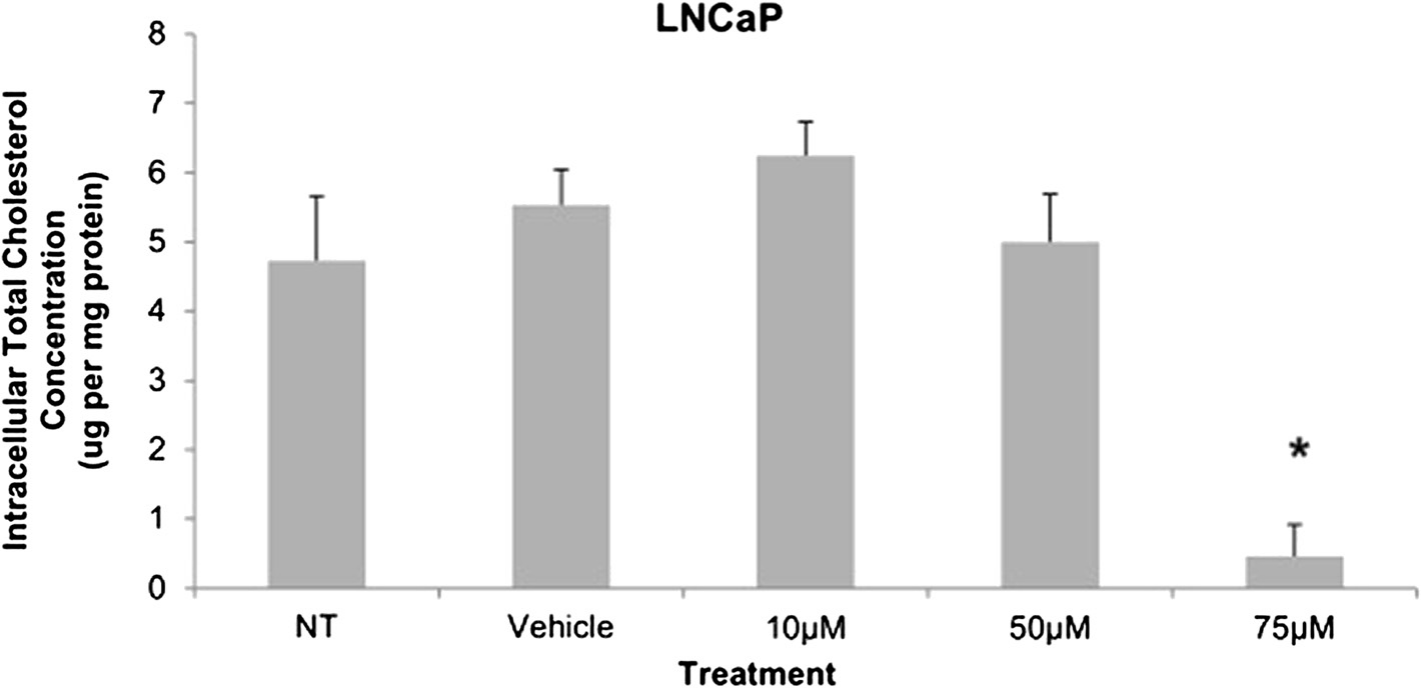
**Total cholesterol concentration in whole cell lysates of LNCaP cells after 48 hour Simvastatin treatment.** Columns, mean (n = 6); bars, ±SEM. *P < 0.05, Vehicle control versus 75 μM.

**Figure 14:**
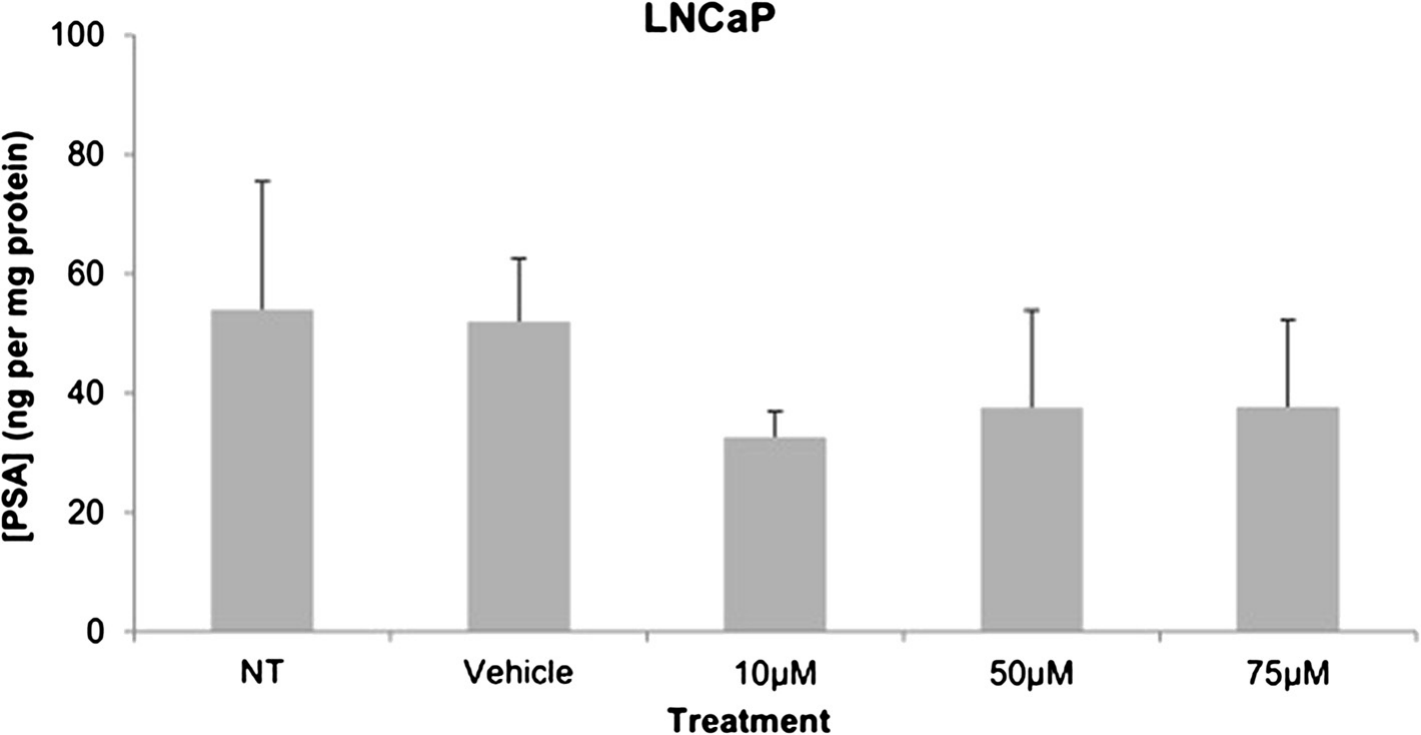
**PSA production demonstrated as secreted PSA levels in the media of LNCaP after 48 hour Simvastatin treatment.** Columns, mean (n = 6); bars, ±SEM.

Cytotoxicity results expressed as mean values normalized to a 100% death control were found to be significantly different at 60 μM, 75 μM, 80 μM, 100 μM, and 250 μM when compared to vehicle control group’s cytotoxicity mean value (Figure 8). Corresponding cell viability results were observed where cellular viability, expressed as fold change from negative control, decreased significantly at 75 μM, 80 μM, 100 μM, and 250 μM, and Triton-X 1% control (Figure 9). The control LNCaP cells showed a cumulative growth curve that was showed a significant difference in the growth pattern of upon 75 μM of Simvastatin treatment compared to vehicle control at Days 5 and 8 (Figure 10). Upon Simvastatin treatments, LNCaP cells displayed a significant reduction in cholesterol synthesis activity is at 75 μM of Simvastatin treatment as shown in Figure 11; however, no significant changes were observed in the HMGCR expression across the Simvastatin doses (Figure 12). The total intracellular cholesterol concentrations, normalized to protein levels (mg), showed a significant reduction in the total cholesterol concentration at 75 μM of Simvastatin compared to vehicle control (Figure 13). The decrease in cholesterol levels corresponded to the decrease in HMGCR protein activity at 75 μM Simvastatin treatment results. Despite the change in cholesterol synthesis activity and total intracellular cholesterol concentration, there were no significant changes observed in the levels of PSA secretion (ng/mL) of LNCaP cells, normalized to the amount of protein (mg), upon Simvastatin treatments (Figure 14).

## Discussion

In the past castration-resistant prostate cancer, now with a median overall survival of 18.4 months with combination therapy of docetaxel and AR blocker MDV3100, was misunderstood as an ‘androgen independent’ form of prostate cancer based on the observations that it is able to recur and more importantly aggressively metastasize in patients treated with androgen deprivation therapy [23]. As mentioned however, many groups have reported the continuous expression of AR and activation of the AR-androgen signaling pathway in castration-resistant prostate cancer through both *in vitro* as well as *in vivo* studies despite the androgen castrated environment, supporting the existence of an intracellular *de novo* androgen synthesis pathway rather than the tumor’s ability to sustain itself despite the lack of androgens [24-27].

HMGCR has been shown to be essential regulator for provision of cholesterol to the androgen synthesis pathway in the steroidogenic tissues of the body [15]. Upon progression to castration-resistance in LNCaP xenograft model, an increase *ex vivo* HMGCR activity has been observed [6]. Also, in castration resistant cell model upon the knockdown of SR-BI transporter a significant increase in HMGCR activity was observed [15]. These finding suggests that castration-resistant cells may adapt to the low androgen environment by increasing cholesterol synthesis to supply cellular needs as well as providing the precursor for *de novo* androgen synthesis. While suggesting an important role of this enzyme in intracellular *de novo* androgen synthesis and potentially suggesting common HMGCR inhibitors such as statins as possible therapeutic agent, the effect of inhibiting HMGCR in a castration-resistant model has not been investigated. The current study aimed to investigate the physiological relevance of one source of cholesterol to the cell, HMGCR, a rate-limiting enzyme in the cholesterol synthesis-mevalonate pathway.

Simvastatin is a commonly used drug to control hypercholesterolemia to prevent cardiovascular disease, and as such epidemiological studies have shown significantly lower serum PSA, tumor volume, and percentage of cancer in radical prostatectomy samples in PCa patients on preoperative statins compared to non-users [28,29]. As mentioned, the effects of statins in prostate cancer models have been extensively studied in cell and animal models as well, reporting a decrease in tumor viability and proliferation in PC-3 *in vitro* model as well as reduction in PSA production in mice *in vivo* model; however, the effects have not been thoroughly investigated in CRPC models [24-27]. The treatment of Simvastatin in androgen-sensitive LNCaP cells confirmed the cytotoxic effects as seen in previous studies (Figure 1). In addition, the inhibition of HMGCR activity via Simvastatin significantly stunted the cellular growth of castration-resistant C4-2 cells in a similar dose-dependent manner as the control LNCaP cells (Figures 1 and 8). The two cell lines were shown to display cellular cytotoxicity, viability, and growth curve patterns that are not significantly different from each other; both cell lines showed nearly 50% reduction in cell viability after 48 hour incubation of 75 μM of Simvastatin (Figures 1, 2 and 3).

Cholesterol is an important precursor molecule in the pathways involved in the production of androgens, which stimulate prostate growth; the changes in growth and proliferation seen in Figures 1, 2 and 3 may be a result of cholesterol synthesis alteration caused by Simvastatin. Upon Simvastatin treatments, C4-2 cells showed significant changes in the HMGCR activity, normalized to protein amount (μg), at 50 μM and 75 μM of Simvastatin (Figure 4). Corresponding to past studies which reported an increase in *ex vivo* HMGCR activity upon progression to castration-resistance in a LNCaP xenograft model, the current study also reported a five-fold increase in basal cholesterol synthesis activity in the no treatment groups of C4-2 cells compared to LNCaP cells as shown in Figures 4 and 11 [6]. Upon examining the HMGCR protein expression levels, Simvastatin treatment did not significantly alter the expression of the HMGCR protein in C4-2 and LNCaP cells (Figures 5 and 12); as a competitive inhibitor of HMGCR, statins are not known to affect the enzyme function via silencing mechanism. Corresponding to the HMGCR activity results, at 75 μM of Simvastatin both cell lines showed a significant decrease in the intracellular cholesterol content, normalized to protein levels (mg) (Figures 6 and 13). However in C4-2 cells, upon 50 μM of Simvastatin, the intracellular cholesterol quantity remained the same despite the decrease in HMGCR activity, which may be an indication of compensatory pathways activation to supply the cell with the necessary cholesterol source via other cholesterol homeostasis pathways despite the inhibition of HMGCR (Figures 4 and 6).

An important indication of AR-androgen pathway activation is the production of PSA, which is produced in response to transcription of genes that contain androgen response elements (AREs). Similar to the PSA observations seen in previously published papers, the basal PSA secretion levels in C4-2 cells was significantly higher; the current study showed an increase that is approximately five magnitude greater in C4-2 cells than the amount secreted by LNCaP cells (Figures 7 and 14) [30]. While previous studies have reported a lack of change in the level of AR protein expression between LNCaP and C4-2 cells, statin use has been shown to decrease the expression and activity of the AR via suppression of the Akt/mTOR signaling pathway [15,30] These finding indicates the existence of a continuous androgen supply, and whether there is a greater total amount of androgens to activate the AR or the ligand-receptor binding interaction is stronger in C4-2 cells is unknown. Furthermore, the inhibition of cholesterol synthesis significantly reduced the secreted PSA levels in C4-2 cells at 50 μM and 75 μM of Simvastatin compared to vehicle control, while no differences were observed in LNCaP cells across the treatment groups implicating that the decrease in cholesterol synthesis has a greater effect in castration-resistant state and supporting the hypothesis of existence and greater reliance on intracellular *de novo* steroidogenesis in CRPC.

In addition to the competitive inhibition of HMGCR, Simvastatin like most other statins may exert cholesterol-independent or pleiotropic effects through direct inhibition of small signaling molecules; by inhibiting HMGCR, statins inhibit cholesterol synthesis as well as the synthesis of isoprenoids which are important lipid attachments for intracellular signaling molecules such as Rho, Rac, and Cdc42 [31,32]. Despite the decreases in HMGCR activity, total intracellular cholesterol, and PSA, if a constant intracellular testosterone level across the Simvastatin treatment groups were observed, it may indicate that the signaling processes are being affected. In the current study, the decrease in PSA secretion may be due to the direct effect of Simvastatin on cholesterol-androgen production and androgen receptor activation and/or indirect effect on the signaling pathways.

The effect of regulating the cholesterol homeostasis pathway to alter the growth of CRPC cells has also been seen in cholesterol influx regulation [15]. Recently scavenger-receptor class B type I (SR-BI) receptor has also emerged as a potential therapeutic target of interest in investigating PCa progression as well as *de novo* androgen synthesis due to the discovery of a significant increase in the SR-BI protein expression in CRPC cells compared to non-castrated LNCaP cells [15]. SR-BI receptor is one of the two major cholesterol influx transporters, mainly uptake of cholesterol esters bound to high density lipoprotein (HDL) via a docking mechanism. It has been shown that down-regulation of SR-BI expression via gene silencing significantly reduced the viability of C4-2 cells, and at a greater extent compared to LNCaP cells [15]. Furthermore, a significant reduction in the amount of PSA secreted by both C4-2 and LNCaP cells was observed when comparing SR-BI knockdown to negative control cells [15]. However, the limitations of the study were that no corresponding differences in intracellular cholesterol and androgen levels were observed. The results from the current study looking at inhibition of cholesterol synthesis via use of Simvastatin reported a significant reduction in cell viability, intracellular cholesterol levels, PSA secretion, and cholesterol synthesis activity in C4-2 cells upon Simvastatin treatments. It would be worthwhile to investigate the inhibition of multiple cholesterol regulatory pathways including cholesterol influx and synthesis using combination treatment of SR-BI silencing and Simvastatin to assess the effect of a combination treatment in CRPC models.

## Conclusion

In summary, the inhibition of HMGCR via Simvastatin lowered the viability of castration-resistant C4-2 cells. Simvastatin’s ability to limit the endogenous supply of cholesterol likely contributes to the effects seen in cell viability.

## Materials and methods

### Materials

Poly-L-lysine (0.01% solution), cholesterol, Triton® X-100, dimethyl sulphoxide, phenylmethylsulfonyl fluoride, protease inhibitor cocktail, Trizma®-hydrochloride, Trizma® base, glycine, lyophilized bovine serum albumin, sodium dodecyl sulfate, ammonium persulfate, tetramethylethylenediamine, sodium hydroxide, sodium chloride, sodium deoxycholate, nonyl phenoxypolyethenyl ethanol (NP-40) and ethylenediamininetetraacetic acid (Sigma-Aldrich, St. Louis, MO, USA), acetic acid [1-14C]- (American Radiolabeled Chemicals, Inc., Saint Louis, MO, USA), cholesterol [1, 2-3H(N)]- (PerkinElmer Inc. Waltham, MA, USA). Scintillation fluid was purchased from MP Biomedicals (Solon OH, USA). RPMI-1640 without phenol red, Hank’s balanced salt solution, 0.25% trypsin-EDTA, penicillin-streptomycin liquid, fetal bovine serum, charcoal-stripped fetal bovine serum, were purchased from Invitrogen (Life Technologies, Invitrogen, Burlington, Ontario). Chloroform, ethyl acetate, hexanes, ethanol, methanol and isopropanol were purchased from Fisher Scientific (Waltham, MA, USA). SuperSubstrate® West Pico Solution was obtained from Thermo Scientific (Rockford IL, USA).

### Cell culture

C4-2 and LNCaP cells were obtained from the Vancouver Prostate Centre. LNCaP cells were used for experiment between the passage numbers of 42-52. The cells were cultured in RPMI-1640 medium without phenol red containing 10% fetal bovine serum and 1% penicillin-streptomycin at 37°C in 5% CO_2_ environment. 72 hours prior to Simvastatin treatment, the cells were seeded in plates in 10% fetal bovine serum supplemented RPMI medium. After the initial 24 hours, the medium was replaced with RPMI medium supplemented with 5% dextran-charcoal stripped medium with restricted androgens to mimic an androgen-castrated environment. Cells were seeded in plates to include vehicle control, Simvastatin treatment, no treatment (NT) and 100% cell lysis (Triton X100, 1%) control wells.

### Cell cytotoxicity and viability

Cells were plated in 96-well plates at a density of 6 × 10^3^ cells per well 72 hours prior to Simvastatin treatment. Treatments and controls were assigned in triplicates in each plate. 50 μL of media was taken from above the cells 48 hours post Simvastatin incubation for LDH cytotoxicity assay. The remaining cells were rinsed with Hank’s Balanced Salt Solution (HBSS) and MTS assay was performed to detect cell viability. Protein determination was performed using BSA assay to normalize the MTS values to protein concentration.

### Cumulative cellular growth

Cells were plated in 6-well plates at a density of 1.8 × 10^5^ cells per well 72 hours prior to Simvastatin treatment. 48 hour post-seeding (Day 0), cells were carefully detached from the plates using 0.25% Trypsin-EDTA, and counted using haemocytometer. The procedure was repeated at 72 hour post-seeding (Day 1), 48 hours post-Simvastatin treatment (Day 3), and 72 hours post-Simvastatin treatment removal (Day 6).

### Cholesterol synthesis (HMGCR activity) assay

C4-2 and LNCaP cells were seeded in 12-well plates at a density of 8 × 10^5^ cells per well 72 hours prior to Simvastatin treatment. Following the Simvastatin treatment for 48 hours, the cells were washed twice with HBSS and labeled with 0.5 μCi of ^14^C-acetic acid prepared in fresh Simvastatin solutions for 3 hours. Cells were washed three times with HBSS following incubation. The lipid content in the samples was extracted using the Bligh Dyer lipid extraction method as previously described, dried under nitrogen, and reconstituted in chloroform [33]. The chloroform samples were separated using thin layer chromatography on silica gel plate in 9:1 hexane to ethyl acetate solution. A 10 mg/mL cold cholesterol sample and an internal extraction control, ^3^H [1,2]-cholesterol loaded at 1 uCi/mL, were included in the thin layer chromatography procedure as controls. After the solution has reached the top, the plate was air-dried and exposed to iodine crystals. The yellow cholesterol bands were excised and reconstituted in scintillation counter fluid to be measured in beta scintillation counter (Perkin Elmer).

### Protein expression

Protein levels were measured in the collected supernatants and known amounts of protein were separated using 4-15% SDS gels. Protein transfer, membranes were blocked and incubated overnight with HMGCR (Upstate Biotech®) and β-actin (Santa Cruz®) 1° antibodies at 1:1000 dilutions. Membranes were then incubated with HRP-labeled 2° antibodies at a 1:3000 dilution to detect the proteins using chemiluminescence detection method.

### Quantification of cholesterol and prostate specific antigen

The Amplex Red Cholesterol assay (Invitrogen) was performed as per manufacturer’s instructions to measure the total intracellular cholesterol amount. 50 μL of cell extracts and cholesterol reference standards were aliquoted in triplicates into a 96-well plate and Amplex Red reaction buffer and working solution were added. Plates were incubated for thirty minutes at 37°C in the dark. Fluorescence was measured using an excitation wavelength of 560 nm and an emission wavelength of 590 nm. Cholesterol concentration was normalized to the amount of protein present in the lysates.

The amount of PSA secreted by the cancer cells was quantified using the PSA ELISA assay (ProClin International). 50uL of reference standards and media from each treatment and control wells were aliquoted in triplicates into a 96-well plate. Following incubations with reaction buffer, enzyme conjugate reagent, and TMB reagent, the optical density was measured at 450 nm and the PSA concentration was normalized to the amount of protein present in the media.

### Statistical analysis

All data are presented as mean + standard error of the mean. Paired t-tests and one-way ANOVA tests (SigmaStat 3.5) were performed to compare the normalized expressions and concentrations between treatment groups. Tukey post hoc tests were done to determine the critical differences, and a difference was considered to be significant if the probability of chance explaining the difference was less than 5% (p < 0.05).

## Abbreviations

ACAT: Acetyl-CoA acyltransferase; ADT: Androgen deprivation therapy; AR: Androgen receptor; CRPC: Castration-resistant prostate cancer; DHT: Dihydrotestosterone; ELISA: Enzyme-linked immunosorbent assay; HMG-CoA: 3-hydroxy-3-methylglutaryl-coenzyme A; HMGCR: 3-hydroxy-3-methylglutaryl-coenzyme A reductase; HSL: Hormone sensitive lipase; LDH: Lactate dehydrogenase; LDLr: Low density lipoprotein receptor; MTS: (3-(4,5-dimethylthiazol-2-yl)-5-(3-carboxymethoxyphenyl)-2-(4-sulfophenyl)-2H-tetrazolium; NT: No Treatment; PCa: Prostate cancer; PSA: Prostate specific antigen; SR-BI: Scavenger receptor Class B Type I; SV: Simvastatin.

## Competing interests

The authors declare that they have no competing interests.

## Authors’ contributions

JK carried out the studies, performed the statistical analysis, and drafted the manuscript. MC and KW conceived of the study, participated in the design of the study, and helped to draft the manuscript. All authors read and approved the final manuscript.
